# Occult Lung Malignancy Presenting With Severe Paraneoplastic Hypercalcemia as an Early Diagnostic Clue in a Patient With Chronic Obstructive Pulmonary Disease (COPD) and Smoking History: A Diagnostic Challenge Mimicking Chronic Infection

**DOI:** 10.7759/cureus.109408

**Published:** 2026-05-21

**Authors:** Nicholsan Jesiah, Nirasha Jayathilake, Manil Peiris

**Affiliations:** 1 University Medical Unit, Teaching Hospital Batticaloa, Batticaloa, LKA; 2 Respiratory Medicine, National Hospital Kandy, Kandy, LKA

**Keywords:** carcinoma, hypercalcemia, infection, parathyroid hormone-related peptide (pthrp), tuberculosis

## Abstract

Non-resolving pneumonia in elderly patients with chronic obstructive pulmonary disease (COPD) and a smoking history poses a significant diagnostic challenge, particularly in tuberculosis-endemic regions where infectious causes are often prioritized. We report a 68-year-old male patient with COPD, type 2 diabetes mellitus, and hypertension who presented with progressive dyspnea, evening low-grade fever, anorexia, and significant weight loss over three months. Initial treatment for presumed infective exacerbation of COPD failed despite antibiotics. Extensive workup for tuberculosis, multiple myeloma, and sarcoidosis was negative. Notably, prominent parathyroid hormone (PTH)-independent hypercalcemia was detected early and persisted, serving as the pivotal diagnostic clue. Contrast-enhanced CT (CECT) chest revealed a left lower lobe mass with endobronchial obstruction, and biopsy confirmed squamous cell carcinoma. The hypercalcemia was presumed to be mediated by tumor-derived parathyroid hormone-related peptide (PTHrP). This case highlights the value of recognizing paraneoplastic hypercalcemia as an early red flag for occult lung malignancy in high-risk patients, even with modest smoking exposure, thereby preventing diagnostic delay due to anchoring on infection.

## Introduction

Non-resolving pneumonia, defined as persistence of pulmonary infiltrates beyond four to six weeks despite adequate antimicrobial therapy, poses a diagnostic dilemma. Differentials include resistant or atypical infections (particularly tuberculosis), malignancy, and noninfectious inflammatory conditions. In tuberculosis-endemic regions, diagnostic anchoring on infectious etiologies frequently delays recognition of underlying malignancy [1, 2].

Lung cancer remains the leading cause of cancer-related mortality worldwide, and squamous cell carcinoma often presents as a central tumor causing endobronchial obstruction and post-obstructive pneumonia that closely mimics infection [3]. Paraneoplastic hypercalcemia occurs in up to 20-30% of malignancies and is strongly associated with squamous cell carcinoma of the lung, primarily due to secretion of parathyroid hormone-related peptide (PTHrP), leading to humoral hypercalcemia of malignancy (HHM) [4, 5].

This case is noteworthy because prominent paraneoplastic hypercalcemia manifested early as the leading biochemical clue that redirected the diagnostic pathway toward malignancy, despite a relatively modest smoking history of only five pack-years, repeatedly negative infectious workups, and the challenges of a tuberculosis-endemic setting. It underscores the importance of maintaining a high index of suspicion for occult lung cancer when PTH-independent hypercalcemia accompanies non-resolving pneumonia.

## Case presentation

A 68-year-old male patient with COPD, type 2 diabetes mellitus, and hypertension presented with progressive exertional dyspnea, intermittent evening low-grade fever, anorexia, and unintentional weight loss over three months. He had a modest smoking history of five pack-years. Physical examination revealed reduced air entry in the left lower lung zone. Chest radiograph showed left lower lobe consolidation (Figure 1).

**Figure 1:**
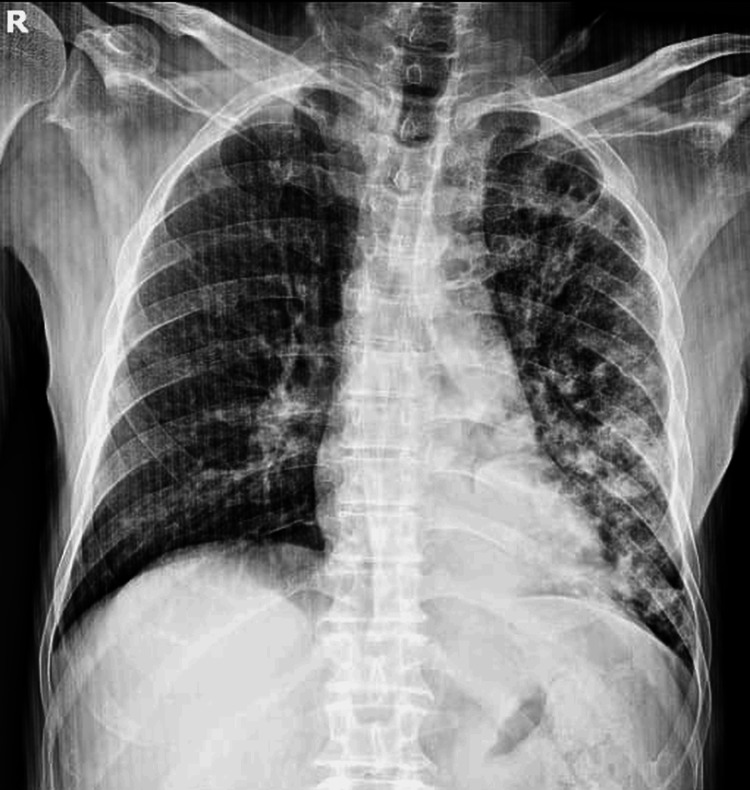
Chest radiograph showing left lower lobe consolidation.

He received two courses of antibiotics and bronchodilators for presumed infective exacerbation of COPD, but symptoms and radiographic findings persisted after four weeks.

Laboratory investigations revealed a markedly elevated erythrocyte sedimentation rate (ESR) of 132 mm/hr. Repeated sputum evaluations for tuberculosis (acid-fast bacilli smear and Xpert MTB/RIF (Cepheid, Sunnyvale, CA, USA) assay) were negative. Screening for multiple myeloma and sarcoidosis was unremarkable. However, hypercalcemia was noted early (corrected serum calcium 2.92 mmol/L) with suppressed intact PTH (10 pg/mL), consistent with PTH-independent paraneoplastic hypercalcemia. Other laboratory parameters are summarized in Table 1.

**Table 1:** Laboratory investigations.

Investigation	Result	Reference range	Interpretation
Corrected serum calcium	2.92 mmol/L	2.10–2.55 mmol/L	Hypercalcemia
Parathyroid hormone (PTH)	10 pg/mL	10–65 pg/mL	Suppressed; suggestive of PTH-independent hypercalcemia
Erythrocyte sedimentation rate (ESR)	132 mm/hr	<20 mm/hr	Markedly elevated inflammatory marker
Serum albumin	3.5 g/dL	3.5–5.0 g/dL	Normal
Serum creatinine	1.10 mg/dL	0.6–1.2 mg/dL	Preserved renal function
Alkaline phosphatase (ALP)	Within normal limits	40–130 U/L	No evidence of increased bone turnover
Urine Bence Jones protein	Negative	Negative	Multiple myeloma less likely
Angiotensin-converting enzyme (ACE) level	Within normal limits	8–52 U/L	Sarcoidosis less likely
Hemoglobin	11 g/dL	11–16 g/dL	Mild anemia
White blood cell count	11 × 10⁹/L	4–11 × 10⁹/L	Upper limit of normal
Platelet count	382 × 10⁹/L	150–400 × 10⁹/L	Normal
Serum phosphate	0.8 mmol/L	0.8–1.5 mmol/L	Low-normal
Liver function tests	Within normal limits	—	No hepatic dysfunction
Sputum acid-fast bacilli smear	Three samples negative	Negative	No microbiological evidence of tuberculosis
Sputum Xpert MTB/RIF assay	Negative	Negative	No evidence of Mycobacterium tuberculosis detected

Contrast-enhanced CT of the chest demonstrated a left lower lobe mass with endobronchial extension (Figure 2).

**Figure 2:**
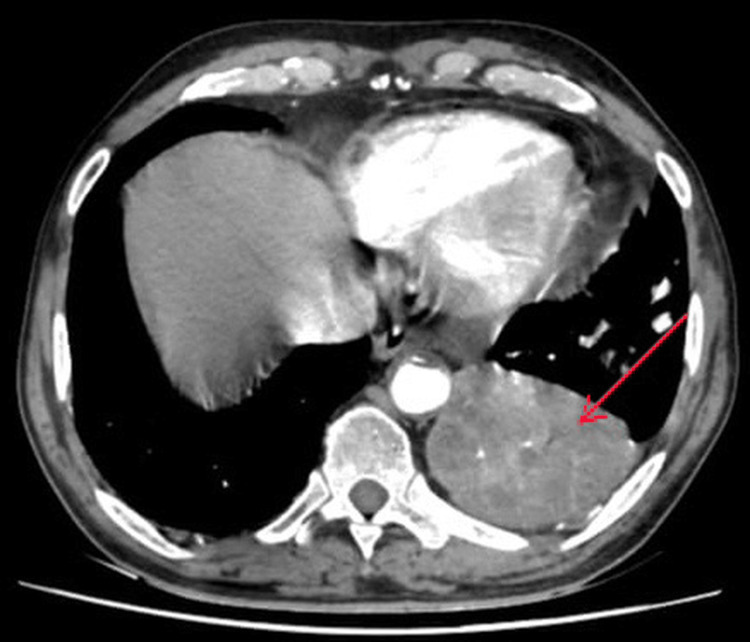
Contrast-enhanced CT chest showing a left lower lobe mass with endobronchial extension. Contrast-enhanced computed tomography (CT) of the chest demonstrates a heterogeneously enhancing soft-tissue mass lesion in the left lower lobe of the lung (indicated by the red arrow).

Flexible bronchoscopy revealed complete obstruction of the left main bronchus by an endobronchial tumor with necrotic debris (Figure 3).

**Figure 3:**
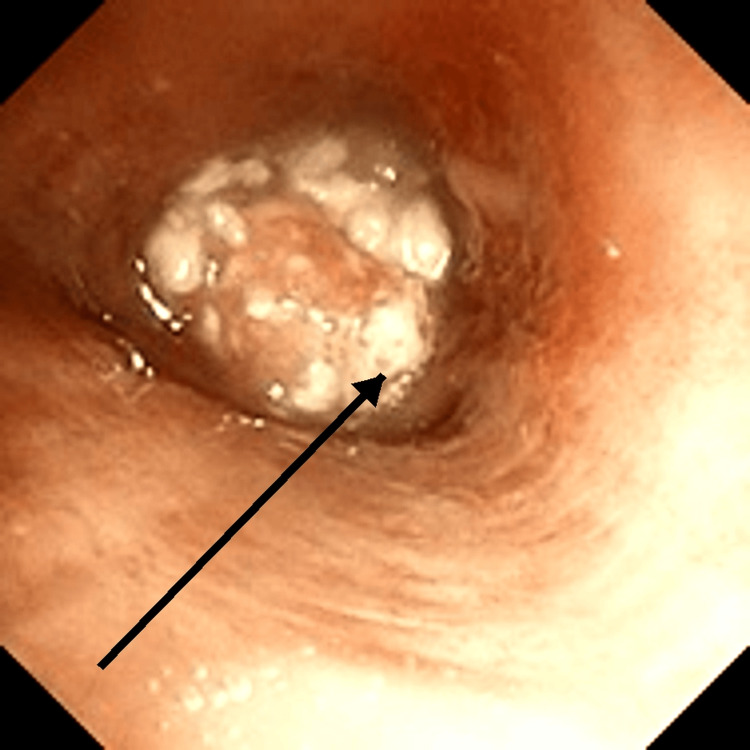
Flexible bronchoscopy showing complete obstruction of the left main bronchus by an endobronchial tumor with necrotic debris. Flexible bronchoscopy demonstrates a polypoidal, irregular endobronchial mass lesion arising within the left main bronchus. The arrow highlights the friable tumor tissue causing near-complete luminal obstruction of the bronchus.

Histopathological examination of the bronchial biopsy confirmed moderately differentiated squamous cell carcinoma with keratin pearls and intercellular bridges (Figure 4). The patient was referred to oncology for staging and multidisciplinary management. Hypercalcemia was managed with intravenous hydration and bisphosphonates, with partial improvement.

**Figure 4:**
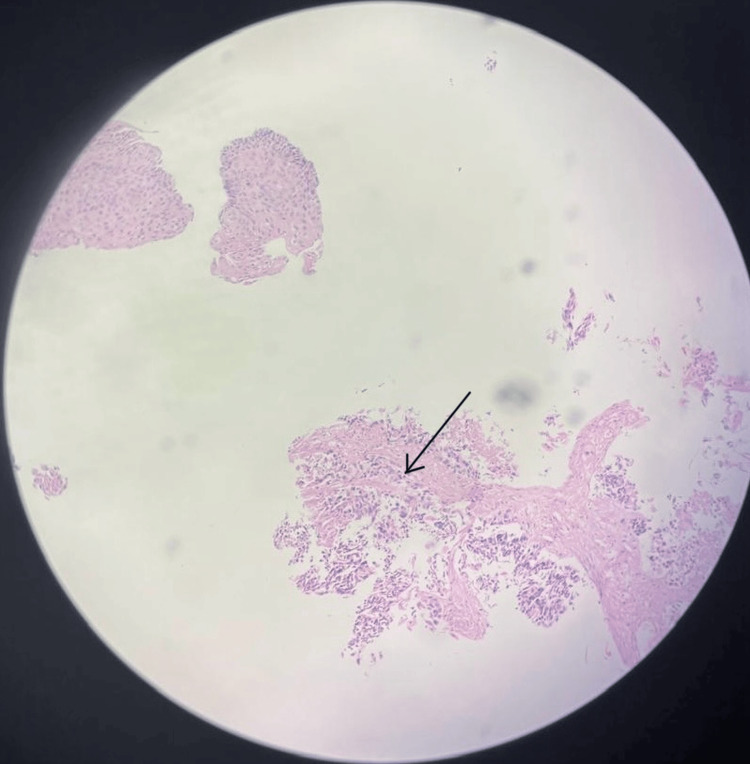
Histopathological examination of the bronchial biopsy demonstrating moderately differentiated squamous cell carcinoma with keratin pearls and intercellular bridges (H&E stain, ×200 magnification). Histopathological examination of the bronchial biopsy specimen stained with hematoxylin and eosin (H&E) demonstrates malignant squamous epithelial cells arranged in nests and sheets. The arrow highlights a keratin pearl, which is formed by concentric layers of eosinophilic keratinized material produced by malignant squamous cells.

## Discussion

This case illustrates the diagnostic utility of prominent paraneoplastic hypercalcemia as an early indicator of occult squamous cell lung carcinoma in a patient with COPD and a modest smoking history presenting with non-resolving pneumonia. In high-risk individuals, lack of response to antimicrobial therapy accompanied by PTH-independent hypercalcemia should prompt early consideration of malignancy [4, 5].

In tuberculosis-endemic settings, clinicians commonly anchor on infectious causes, which can significantly delay the diagnosis of lung cancer. Central squamous cell tumors frequently cause endobronchial obstruction and post-obstructive consolidation that radiologically and clinically mimic chronic infection [1, 2]. In this patient, the early and prominent hypercalcemia with suppressed PTH served as the pivotal clue that accelerated contrast-enhanced imaging and bronchoscopic evaluation despite negative infectious workups.

Hypercalcemia of malignancy in squamous cell lung carcinoma is primarily mediated by tumor-derived PTHrP, which increases osteoclastic bone resorption and renal calcium reabsorption while promoting phosphaturia. This mechanism explains hypercalcemia and hypophosphatemia even without extensive bone metastases [4, 5]. Nonspecific symptoms such as anorexia and fatigue overlap with those of COPD exacerbation or infection, further complicating diagnosis. Hypercalcemia may precede obvious radiologic detection of the tumor and is generally associated with more advanced disease and poorer prognosis.

Similar cases of PTHrP-mediated hypercalcemia in squamous cell lung cancer have been reported. Nemr et al. (2017) described concurrent elevation of PTHrP and calcitriol [6]. Prakash et al. (2023) reported a rare case of predominantly calcitriol-mediated hypercalcemia with only minimally elevated PTHrP [7]. A more recent report highlighted simultaneous PTHrP- and calcitriol-mediated hypercalcemia in a case with histologic transformation [8]. However, most published cases describe hypercalcemia in the context of known advanced disease or heavier smoking burdens. The uniqueness of our case lies in the early, prominent hypercalcemia acting as the primary diagnostic red flag in a patient with relatively light tobacco exposure (five pack-years) and in a resource-constrained, TB-endemic environment where infectious etiologies dominate initial reasoning. This reinforces the need to consider paraneoplastic syndromes even when classic risk profiles appear less pronounced.

A structured diagnostic approach integrating clinical risk factors, therapeutic non-response, and biochemical red flags such as PTH-independent hypercalcemia is essential to avoid delays. In practice, unexplained hypercalcemia in a smoker with non-resolving consolidation should lower the threshold for early contrast-enhanced CT and bronchoscopy, even in TB-endemic areas [2, 9, 10].

## Conclusions

Prominent paraneoplastic hypercalcemia can serve as a valuable early diagnostic clue for occult lung malignancy in patients with COPD and smoking history who present with non-resolving pneumonia. In tuberculosis-endemic regions, clinicians should maintain a high index of suspicion for malignancy when PTH-independent hypercalcemia persists despite negative infectious evaluations. Early recognition of this paraneoplastic phenomenon, followed by prompt cross-sectional imaging and tissue diagnosis, facilitates timely oncology referral and may improve clinical outcomes.
